# Microbial Diversity of Emalahleni Mine Water in South Africa and Tolerance Ability of the Predominant Organism to Vanadium and Nickel

**DOI:** 10.1371/journal.pone.0086189

**Published:** 2014-01-22

**Authors:** Ilunga Kamika, Maggie N. B. Momba

**Affiliations:** Department of Environmental, Water and Earth Sciences, Faculty of Science, Tshwane University of Technology, Pretoria, Gauteng, South Africa; RMIT University, Australia

## Abstract

The present study aims firstly at determining the microbial diversity of mine-water collected in Emalahleni, South Africa and secondly isolating and characterizing the most dominant bacterial species found in the mine water in terms of its resistance to both V^5+^ and Ni^2+^ in a modified wastewater liquid media. The results revealed a microbial diversity of 17 orders, 27 families and 33 genera were found in the mine-water samples with *Marinobacteria* (47.02%) and *Anabaena* (17.66%) being the most abundant genera. Considering their abundance in the mine-water samples, a species of the *Marinobacter* genera was isolated, identified, and characterised for metal tolerance and removal ability. The MWI-1 isolate (*Marinobacter* sp. MWI-1 [AB793286]) was found to be closely related to *Marinobacter goseongensis* at 97% of similarity. The isolate was exposed to various concentrations of Ni^2+^ and V^5+^ in wastewater liquid media and its tolerance to metals was also assessed. The MWI-1 isolate could tolerate V^5+^ and Ni^2+^ separately at concentrations (in terms of MIC) up to 13.41±0.56 mM and 5.39±0.5 mM at pH 7, whereas at pH 3, the tolerance limit decrease to 11.45±0.57 mM and 2.67±0.1 mM, respectively. The removal of V^5+^ and Ni^2+^ in liquid media was noted to gradually decrease with a gradual increase of the test metals. A significant difference (p<0.05) between V^5+^ and Ni^2+^ removal was noted. *Marinobacter* sp. MWI-1 achieved the maximum permissible limit of 0.1 mg-V^5+^/L prescribed by UN-FAO at 100 mg/L, while at 200 mg/L only V^5+^ was removed at approximately 95% and Ni^2+^ at 47%. This study suggests that mine-water indigenous microorganisms are the best solution for the remediation of polluted mine water.

## Introduction

Mine water remains one of the major problems of concern, not only in South Africa, but also worldwide. This is due to its environmental, socio-economic and public health impacts [1]. It is mostly characterised by extreme pH (acidity or alkanity), high salinity levels, high concentrations of SO_4_^2−^, Al and several other toxic metals such as Fe, Cd, Co, Cu, Mo, Zn, Ni, V and sometimes even radionuclide [2]. In South Africa in particular, mining activities have a long history and have played a major role in both economic development and environmental pollution countrywide [3]. Although significant progress has recently been made in South Africa to address mine water management, environmental pollution due to the disposal of untreated mine water still remains [1]. Microorganisms, due to their ubiquitousness, have been viewed as one of the best ways to deal with this problem. Due to their ability to survive, grow and reproduce in such harsh environments, an interest in microorganisms was aroused among researchers worldwide [4]. Nevertheless, their presence in extreme environments such as mine water affects their species diversity [5]. Wang and co-workers [5] have pointed out that extreme conditions can be defined by levels of environmental factors, the effects of which pose difficulties for the survival of specific taxa or all taxa. In addition, both Johnson and Hallberg [6] and Imarla et al. [7] have also reported that microbial community composition is largely bound to geochemical parameters such as pH and metal ion concentrations. As a result, microorganisms isolated from such environments are considered to be a valuable tool in the treatment of highly polluted mine water. A number of techniques such as culture-dependent and culture independent techniques have been developed and used to study microbial diversity of water, wastewater, soil and air [8], [9]. While several microorganisms are not able to readily grow in pure culture, the culture-independent approach has seen its apogee for the simple reason that this method has the advantage of directly profiling microbial populations present in specific ecosystems straight from the environmental samples [10], [11]. Although metal pollution is a major concern worldwide and also in South Africa, the microbial diversity of the mine water in the latter country has not been fully examined. This study is one of a few to describe the microbial diversity present in the water at the vanadium mine in Mpumalanga, South Africa. Moreover, the discovery of new microorganisms in harsh environments has provided some knowledge on the understanding of microbial biosynthetic processes which enhance the bioremediation of contaminated environments [12]. The present study aims at firstly assessing the bacterial diversity of mine water collected from a vanadium mine in South Africa and secondly isolating and characterizing the most dominant bacterial species found in the mine water in terms of its resistance to both V^5+^ and Ni^2+^ in a modified wastewater liquid media.

## Materials and Methods

### Study area and mine water sample collection and preparation

Mine water samples of 500 mL (a total of 48 samples) were collected in a sterile plastic sampling bottle (500 mL) on a weekly basis (with 4 samples per week) between September and November 2012 from the effluent of the vanadium mine in Emalahleni, Mpumalanga, South Africa (25°50′′26.4′ and 29°09′′ 09.9′). No specific permit was needed for the collection of the wastewater samples in the described sample area and this study did not involve endangered or protected species. However, an official letter from the University was submitted to Mr. Ajith Ramnarain (Process Development Manager) to assist us with the collection of wastewater samples. Samples were kept in a cooler box (4°C) while being conveyed to the laboratory for microbial and physicochemical analysis. For microbial analysis, 10 mL of samples (a total of 12 samples) collected at the second week of each month were centrifuged at 10 000 ×g for 10 min and the pellets were thereafter kept under −20°C until analysis, for no more than 4 h. In preparation for the chemical analysis process, samples collected throughout the experiment were allowed to settle for 2 h prior to filtration and No.1 filter papers (Whatman) were used. The profile of the filtered samples was determined in terms of the chemical oxygen demand (COD), dissolved oxygen (DO), pH and chemical contents (metals, semi-metals and non-metals). The COD concentration was measured using closed reflux methods as described in the standard methods of APHA [13], while the pH and the DO were analysed using a pH probe (Model: PHC101, HACH) and DO probe (Model: LDO, HACH), respectively. Chemical elements were determined using the Inductively Couple Plasma Optical Emission Spectrometer (ICP-OES) (Spectro Arcos, Kleve Germany). The limits of detection (LOD) varied between 10–60 µg/L depending on the elements.

### DNA extraction, PCR amplification of purified DNA and pyrosequencing

The cell pellets [10 mL] harvested by centrifuging the mine water were re-suspended in a 1× TE buffer (pH 8.0). An aliquot [10 µL] was examined for protozoan species under a light microscope (Axiovert S100, Carl Zeiss, Germany) at ×100 to ×400 magnification. Bacterial DNA was extracted with the ZR Fungal/Bacterial DNA KitTM (ZYMO Research, Pretoria, South Africa) according to the procedures provided by the manufacturer. The PCR reaction was performed on the extracted DNA samples using universal primers 27F [5′AGRGTTTGATCMTGGCTCAG3′] and 1492R [5′GGTTACCTTGTTACGACTT3′] [14]. A PCR reaction mixture of a total volume of 50 µL, containing 19 µL Nuclease-free water, 25 µL 2× Dream Taq™ PCR master mix (10×Dream Taq™ buffer, 2 µM dNTP mix and 1.25 U Dream Taq™ polymerase), 2 µL of each PCR primer [10 µM] (synthesised by Inqaba Biotechnologies Industry, Pretoria) and 2 µL of genomic DNA [50 ng/µL] was prepared in a 200 µL PCR tube. The amplification was carried out in a thermal cycler (MJ Mini™ Personal Thermal Cycler, Biorad, SA) and consisted of 30 cycles of 1 min each at 94°C of denaturation, 30 s at 58°C of annealing step, 1 min of extension step at 72°C followed by the final extension step of 72°C for 10 min and cooling to 4°C.

The PCR product [10 µL] was analysed using 1% (m/v) agarose gel (Merck, SA) stained with 5% of 10 mg/mL ethidium bromide (Merck, SA) and the correct band size (approximately 1500 bp) was excised. To amplify variable regions (V1-3) of the bacterial 16S rRNA gene, the DNA was recovered from the gel slices by using the GeneJET™ gel extraction kit (Fermentas); thereafter, it was re-amplified with primers A1.4 [5′**CGTATCGCCTCCCTCGCGCCATCA**tctctatgcgAGRGTTTGATCMTGGCTCAG3′] and B1 [5′CTATGCGCCTTGCCAGCCCGCTCAGGTATTACCGCGGCTGCTG3′*] [14]. These primers contained the appropriate adaptor and barcode sequences that were necessary for running the samples on the GS-FLX-Titanium (Roche). The PCR reaction was analysed as described previously, but with an annealing temperature of 50°C as reported by Tekere et al. [8]. The entire PCR product was loaded onto a 1% agarose gel and the correct band size (500 to 600 bp) was excised from the gel and subsequently purified as previously mentioned. The DNA concentrations were quantified by using a Nanodrop spectrophotometer (Nanodrop2000, Thermo Scientific, Japan). The samples were pooled at equal concentrations of the filtration and biofilm samples. The pooled samples were sequenced on the GS-FLX-Titanium series (Roche) at Inqaba Biotechnology Industries, South Africa. Sequences of not less than 200 pb were classified using the online Ribosomal Database Project (RDP) naive Bayesian Classifier, Version 2.4, December 2012, which is assigned to the taxonomical hierarchy: RDP training set 10, based on nomenclatural taxonomy and Bergey's Manual with a confidence threshold of 95%.

### Tolerance limits of test organisms to vanadium and nickel

#### Microbial isolation

Due to the abundance of *Marinobacter* species in the collected mine water samples, the *Halomonas elongata* (HMC) medium was used [15]. The medium was prepared as follows: 7.5 g/L casamino acids, 5 g/L peptone, 1 g/L yeast extract, 50 g/L NaCl, 20 g/L MgSO_4_. 7H_2_O, 3 g/L sodium citrate, 0.5 g/L K_2_HPO_4_, 0.05 g/L FeSO_4_(NH_4_)_2_SO_4_ either with or without 2% agar and pH adjusted at 7.2±0.2. After the medium preparation, it was autoclaved and incubated overnight at 37°C to check for any contamination. Only media which showed no growth were used. The bacteria isolation was achieved by inoculating 1 mL of mine water into the HMC broth for 48 h and thereafter placed on HMC agar. A single colony was selected and serially streaked onto the HMC agar for purification. Pure isolates (MWI-1) were inoculated into the HMC broth to obtain the required bacterial concentration used during the experiment.

#### Modified wastewater liquid media preparation

Domestic wastewater samples were also collected from the effluent (before disinfection) of the Daspoort wastewater treatment plant in Pretoria. This was firstly screened and only samples with very low metal concentrations [<0.01 mg/L] were used for the preparation of the modified wastewater liquid media. d-glucose anhydrate [2.5 g/L], MgSO_4_·7H_2_O [0.5 g/L] and KNO_3_ [0.18 g/L] were added to the filtrate to serve as a carbon source and nutrient supplement for the culture media [16]. The test metal used in the experimental study was of analytical grade, purchased from Sigma Aldrich (Cape Town, South Africa). Sodium meta-vanadate anhydrous (NaVO_3_) and nickel nitrate [Ni(NO_3_)_2_] were used as a source of V^5+^ and Ni^2+^ ions, respectively. The stock solution of V^5+^and Ni^2+^ at a concentration of 2 000 mg/L were prepared using deionised water. From this solution, aliquots of specific volume, corresponding to the final V^5+^ and Ni^2+^ concentrations ranging from 50 mg/L to 800 mg/L (increasing at a geometric scale of 50 mg/L), were added to a 250 mL flask containing the wastewater-mixed liquid medium so as to obtain a final volume of 150 mL. As the optimal growth of Marinobacter goseongensis has been reported occurring at pH 7.5 [17], the pH of the mixed liquid media was adjusted at 7.3±0.3 using 1.0 M HCl and 1.0 M NaOH (Merck, SA). Additionally, a parallel experiment using V^5+^ and Ni^2+^ (1∶1, 2∶1, 1∶2, v:v) concomitantly, was performed. ICP-OES was used to confirm the metal concentrations in the wastewater-liquid media. The culture medium was autoclaved at 121°C for 15 min and cooled down to room temperature prior to use. To check the sterility of this medium, 1 mL aliquot was plated onto the sterile bacteriological agar and incubated at 37°C for 24 h; media indicating any microbial growth were not included in the experimental study. Only flasks containing the sterile media were inoculated with a known population of the respective test organism isolates. In order to mimic the environmental condition, a parallel experiment was carried out at a pH value of 3.

#### Temperature and metal-tolerance characterisation

Prior to assessing the metal tolerance ability of the isolates, the optimum growth temperature of MWI-1 [AB793286] was determined by incubating the isolates at various temperatures (25°C, 30°C and 35°C) in the HMC broth. Afterwards, a series of experiments were conducted in 250 ml Erlenmeyer flasks containing 150 mL of the modified wastewater-liquid media. These series of experiments had one positive control and one negative control. The positive control flask contained the free metals-liquid media medium, while the negative control contained the wastewater-liquid media with the highest concentration [1 000 mg/L] of either V^5+^ or Ni^2+^. Sample flasks as well as those containing the positive controls were inoculated with the isolates (approximately 100 cfu/mL). All the inoculated flasks and the controls were initially incubated at 30°C±2°C and aliquots were taken every day for 4 days. The median lethal concentration (LC_50_) of the test metal for each of the test microbial isolates was determined as described by previous investigators [18]–[21]. The minimum inhibitory concentration (MIC) of the test metal (referring to the smallest concentration of an antimicrobial agent necessary to inhibit growth of microorganisms) was determined according to Shirdam et al. [22]. MIC values were noted when the isolates failed to grow on the plates. After incubation, the microbial isolates were classified as sensitive or tolerant to Ni^2+^ according to the inhibition of growth cells.

#### Effect of pH on the tolerance limits of test organisms

To check the effect of pH on the tolerance limits of bacterial isolate to nickel or vanadium, the isolate was inoculated in mixed liquid media containing the smallest concentration of Ni^2+^ or V^5+^ necessary to inhibit their growth (MIC). The experimental study was conducted at a constant temperature of 30°C in a shaking incubator at a speed of 100 rpm. During each sampling regime, aliquot samples were taken every 24 h for 4 days for microbial estimation.

#### Molecular characterization of the isolate for metal-tolerance ability

In order to assess the ability of the bacterial isolate to tolerate Ni^2+^ and V^5+^ toxicity, its molecular characterisation on metal-tolerance ability was performed by the amplification of the *cnrB2, van2, nccA* and *smtAB* genes that encode for cobalt-nickel-cadium-vanadium resistance as well as, using specific primers (Table 1). The PCR amplification of target genes were done in a thermal cycler (MJ MiniTM Personal Thermal Cycler, Biorad SA) using 200-μL PCR tubes and a reaction mixture volume of 50 μL. The reaction mixture consisted with 25 μl 2× Dream Taq™ PCR master mix (10× Dream Taq™ buffer, 2 μM dNTP mix and 1.25 U Dream Taq™ polymerase), 2 μl of each PCR primer [10 μM] (synthesised by Inqaba Biotechnologies Industry, Pretoria, South Africa) and 2 μl of genomic DNA [50 ng/μl] and was made up 50 μl with ultra-pure nuclease-free water [19 µL]. The following amplification conditions were used: denaturation of template DNA at 94°C for 2 min, followed by 30 cycles of denaturation at 94°C for 1 min, annealing of template DNA for 30 s at specific temperature (Table 1) and an extension time of 1 min at 72°C for the primers. After the last cycle the samples were kept at 72°C for 10 min to complete the synthesis of all the strands and a cooling temperature of 4°C was applied. The PCR product [10 µL] was analysed using 1% (m/v) agarose gel (Merck, SA) stained with 5% of 10 mg/mL ethidium bromide (Merck, SA) and electrophoresed to determine the product size, which was visualised under UV light in an InGenius L Gel documentation system (Syngene).

**Table 1:** Primers targeting some genes encoding metal-resistance in microbes.

Genename	Sequence forward (5′–3′)	Sequence reverse (5′–3′)	Annealing temperature
*nccA*	ACGCCGGACATCACGAACAAG	CCAGCGCACCGAGACTCATCA	57°C
*van2*	CAAGTTCGTCGTCAACTT	CACTCGAGACAGGTATCA	30°C
*smtAB*	GAT CGA CGT TGC AGA GAC AG	GAT CGA GGG CGT TTT GAT AA	56°C
*cnrB2*	TACTGGCGATGTACTCGC	GAAGGTATTACGGGTGGC	55°C

#### Sequencing 16S rRNA

Prior to sequencing, the bacterial DNA was extracted and amplified using the universal primers as stated above. The amplified PCR products of approximately 1 500 bp were purified using a DNA clean and concentrator-25 kit (Zymo Research, SA). The concentrated DNA samples were then stored under −20°C and dispatched to Inqaba Biotechnology Industries (Pretoria, South Africa) for sequencing. The GS Junior-454 Sequencer (Roche) was used to sequence the DNA sample. To carry the phylogenetic analysis, a partial 16S rRNA gene sequence (16S rDNA) from the MWI-1 isolate was compared with other Alteromonadaceae that were available in the database. The 16S rDNA sequences were aligned using CLUSTAL2X and then the phylogenetic tree was constructed with the use of the MEGA 5 computed package. The confidence level of the phylogenetic tree topology was evaluated with a generation of 100 bootstrap sets.

#### Effect of metal ions on cell morphology

To determine the effect of V^5+^ and Ni^2+^ on the cell morphology, the MWI-1 incubated for 24 h in metal solution of V^5+^ or Ni^2+^ [50 mg/L] and the negative control (MWI-1 not exposed to metal ions) were centrifuge sample [10 mL] at 8, 000×g at 4°C for 5 minutes. Microbial cell was washed twice with 0.1 M phosphate buffered saline and fix overnight in 2% glutaraldehyde [prepared in 0.1 M PBS]. Pellets were dehydrated through an ethanol series from 10% to absolute, and for each series samples were held for 30 minutes. Samples were placed on a brass stub, sputter-coated with gold and examined by SEM.

For the Infrared spectra of the treated and untreated biomass were obtained using a Fourier transform infrared spectrometer (FTIR BOMEM MB 104). Prior of analysis, the biomass was centrifuged [10 mL] at 8, 000×g at 4°C for 5 minutes. The pellets were washed twice with 0.1 M phosphate buffered saline, dehydrated through an ethanol series starting from 50% to absolute and thereafter dried. After drying, 5 mg of bacterial cells were encapsulated in 150 mg of KBr. For analysis, all the infrared spectra were recorded over the range of 4000 to 500 cm^−1^.

### Statistical Analyses

The data were statistically analysed using the Stata computer software (version: STATA V12, STATA Corp. LP, 2012). Analysis of variance (ANOVA) was performed to compare the average percentage abundance between different bacteria. The Tukey HSD pairwise comparisons were also performed to see which bacteria where different in terms of the average percentage. The non-parametric Kruskal Wallis test was performed to compare the rank sum of the tolerance rate of the bacteria between V^5+^ and Ni^2+^. The ordinary linear regression and hierarchical linear model were used to compare the average percentage of metal removed by the bacterium between the three treatments, which are mixed, V^5+^ and Ni^2+^. Another statistical multivariate analysis was performed to check the difference between die-off rates at different pH-values when considering the type of test metals and incubation time. Every statistical analysis performed assumed that the observations were dependent between each other. For each parameter, data analysis was repeated assuming the observations are independent. The interpretation was performed at a two-sided 95% confidence limit. Each experimental study or analysis was performed in triplicate except for metal uptake and tolerance limits which were performed in quintuplicate.

### Nucleotide sequence accession number

The 16S rRNA gene sequence of the isolate MWI-1 (*Marinobacter* sp. MWI-1) has been deposited in DNA Data Bank of Japan (DDBJ) Nucleotide sequence database and is available from the above database (http://www.ddbj.nig.ac.jp) as well as GenBank nucleotide database at the NCBI website (http://www.ncbi.nlm.nih.gov) under accession number AB793286.

## Results

### Mine water profile

The chemical profile of the mine water samples is presented in Table 2. Several chemical elements were found in the samples with Zn, Cu, Mn, U, Hg, nitrate and phosphorus not exceeding 10 mg/L. Besides sodium (3429.08–6426.48 mg/L), which showed the highest concentration, Ni, V, Fe, Ca, Mg and K were also found to be the most abundant chemical elements in the mine water samples and their concentrations ranged between 11.37 mg/L and 437.46 mg/L. The mine water samples indicated high acidity with pH ranging from 2.34 to 3.78, whereas the temperature, the conductivity and the COD had values ranging between 12.17°C and 13.80°C, 397 µ/Sm and 645 µ/Sm and 529.27 mg/L and 661.54 mg/L, respectively. Although chemical contents in the mine water throughout the sampling period appeared to be different in terms of concentrations, no significant difference (p>0.05) was noted. In contrary, there was only significant difference in the mean concentration between Na and Ni (p<0.05) when adjusting for the time, and the interaction of the time and the chemical. Specifically, Na was on average 5021.8 mg/L (4493.601 to 5549.972) more concentrated than Ni.

**Table 2:** Profile of mine water samples collected from the vanadium mine, South Africa (n = 3).

	Ni [mg/L]	V [mg/L]	Zn [mg/L]	Cu [mg/L]	Mn [mg/L]	U [mg/L]	Fe [mg/L]	Hg [mg/L]
September	31.99±4.25	434.45±67.30	0.24±0.04	5.53±0.03	2.19±0.12	1.58±0.56	13.21±2.38	3.95±0.27
October	22.07±1.68	437.46±53.28	4. 41±1.08	1.51±0.01	6.34±0.18	1.57±0.09	18.11±1.69	4.64±1.28
November	19.79±3.25	420.94±17.64	2.25±0.67	7.52±0.67	3.12±0.05	1.60±0.21	11.37±3.94	2.76±0.06
	**Ca [mg/L]**	**Mg [mg/L]**	**Na [mg/L]**	**K [mg/L]**	**Cr [mg/L]**	**Co [mg/L]**	**As [mg/L]**	**Pb [mg/L]**
September	518.37±37.29	217.65±1.25	5413.85±97.69	216.32±51.69	0	0	0	0
October	507.91±17.29	213.46±4.98	6426.48±43.69	240.93±1.39	0	0	0	0
November	496.28±29.64	206.80±57.37	3429.08±131.5	238.4±15.37	0	0	0	0
	**DO [mg/L]**	**NO_3_^−^** **[mg/L]**	**PO_4_^3−^** **[mg/L]**	**pH** **[pH units]**	**Temperature** **[°C]**	**Conductivity** **[µ/Sm]**	**COD [mg/L]**	
September	5.67±0.22	4. 14±1.98	2.17±0.05	2.3±0.02	13.40±0.07	533.00±2.59	661.54±67.11	
October	5. 73±0.11	2.08±0.15	5.12±0.01	3.8±0.25	12.17±0.05	397.00±1.15	583.61±13.51	
November	5.63±0.04	1.48±0.37	3.07±0.12	3.2±0.02	13.80±0.01	645.00±2.22	529.27±53.25	

### Microbial diversity in mine water

**Figure 1:**
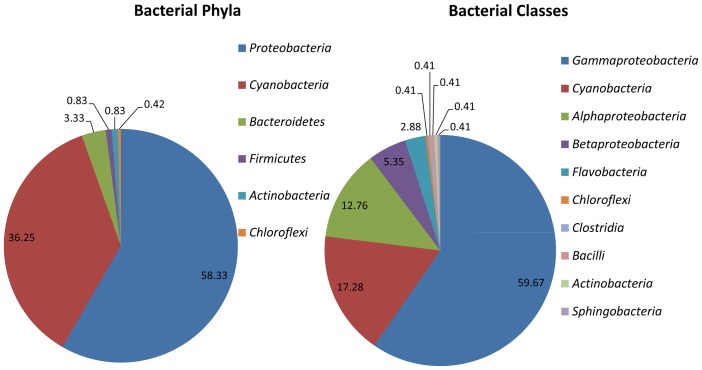
Relative abundance and diversity of bacterial phylum and classes in South African mine water.

The microbial community structure of mine water samples was determined using the 16S rRNA gene amplicon pyrosequencing method which targeted one DNA region (V1–3); respective sequences are summarised in Table 3. A total of 2 047 sequences were identified in the mine water and only sequences with a similarity of 95% to 100% to the species in the database were used. To determine the abundance of each taxon, the number of sequences from a particular taxon was plotted against the total number of sequences used. The bacterial phylum and classes in the South Africa mine water appeared to be less diverse (Figure 1). A total of 6 phyla with 10 classes were identified in the mine water samples. Among these, it is evident that the phyla Proteobacteria (58.33%) and Cyanobacteria (36.25%) were the most abundant, followed by the Bacteriodetes (3.33%), *Firmicutes* (0.83%), *Actinobacteria* (0.83%) and *Chloroflexi* (0.42%). For bacterial classes, *Gammaproteobacteria* (59.67%) was the most predominant in the mine water sample and this was followed by *Cyanobacteria* (17.28%), *Alphaproteobacteria* (12.76%), *Betaproteobacteria* (5.35%) and *Flavobacteria* (2.88%). The rest of the bacterial classes represented a percentage of abundance of less than 0.5%.

**Table 3:** Summary of pyrosequencing data from mine water samples.

Sequence	V 1–3
Number of sequence	2047
Total length of sequences [bp]	769 890
Average length of sequences [bp]	400

**Figure 2:**
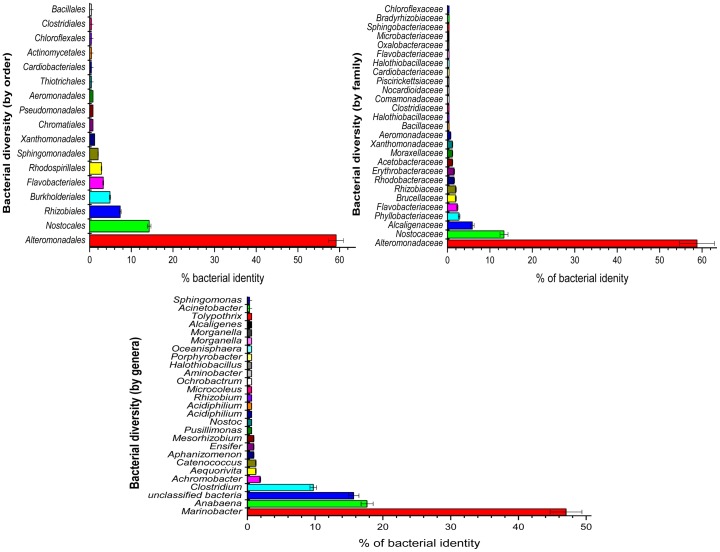
Composition of the bacterial orders, family and genera detected in the mine water with sequences of the variable region V1–3 of the 16S rRNA genes.

In the 6 phyla and 10 classes, a high diversity in terms of bacterial orders, families and genera were observed in the mine water samples (Figure 2), with a total of 17 orders, 27 families and 33 genera being found. Of the orders, *Alteromonadales* (59.18%) was the most abundant followed by *Nostocales* (14.29%) and the rest that revealed less than 10% each. In addition, *Alteromonadaceae* (58.82%) and *Nostocaceae* (17.66%) were the most abundant families present with *Marinobacteria* (47.02%) and *Anabaena* (17.66%) being the most abundant genera. Of the total genera found in the mine water samples, unclassified bacteria (15.67%) were also observed in great abundance when compared to other genera. This was confirmed by a statistical test performing analysis of variance to compare the average percentage abundance between different bacteria. The test is significantly different at a 0.05 significance level. Furthermore, the Tukey HSD pairwise comparisons were also performed to see which bacteria where different in terms of the average percentage abundance and revealed that *Marinobacteria* was on average 31% more abundant than *Anabaena* in the water samples. Similar observation was noted with other bacteria when compared to *Marinobacter* (average ranging from 38–46%).

### Tolerance limits and metal-removal ability of bacterial species

#### Growth curve of the mine water isolates (MWI-1) at different temperatures in metal-free media

**Figure 3:**
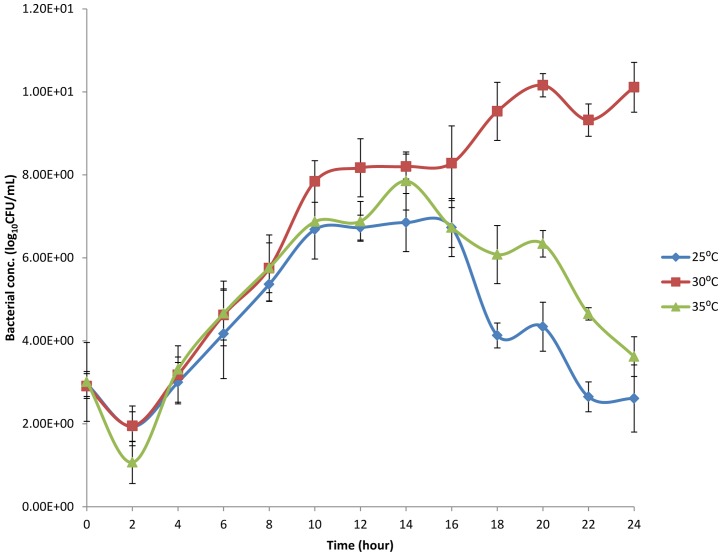
Growth curve of MWI-1 in a metal-free medium (HMC broth) inoculated at different temperatures (25°C, 30°C, 35°C) at pH 7.2±0.2 for 24 h.

Due to their predominance, the genera *Marinobacter* were isolated from the mine water samples and assessed for their possible ability to resist V^5+^ and Ni^2+^. Figure 3 illustrates the growth curves of the MWI-1 in a metal-free medium (HMC broth). The MWI-1 revealed a lag-phase between time 0 to 2 followed by an exponential phase from time 2 h to 8 h when inoculated at 25°C, 30°C and 35°C with bacterial counts of 7 logCFU/mL, 8 logCFU/mL and 7 logCFU/mL, respectively. At 30°C, MWI-1 indicated a second exponential growth [10 logCFU/mL] from time 16 h to the end of the experiment, whereas a death phase was observed at 25°C and 35°C, repectively.

**Figure 4:**
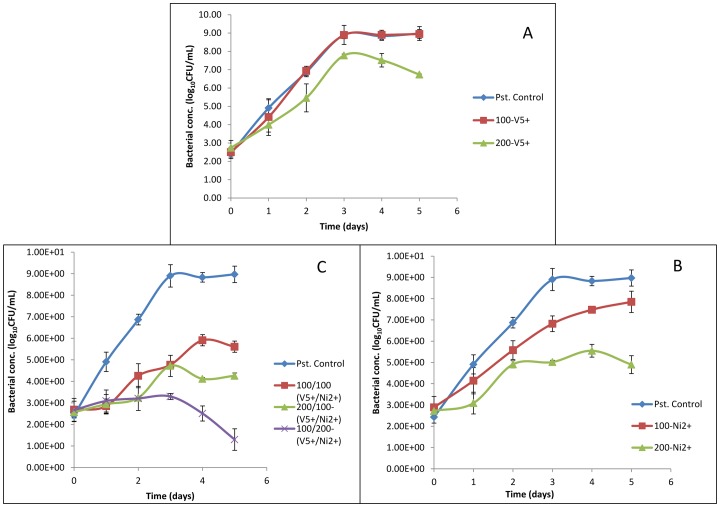
Growth performance of MWI-1 in a medium containing either V^5+^ (A) or Ni^2+^ (B) or both (B) at 100 mg/L and 200 mg/L, 30°C, pH 7.2±0.2.

As MWI-1 grew very well at 30°C, its tolerance to V^5+^ and Ni^2+^ in the modified liquid media was tested at the said temperature and at pH 7.2±0.2. Figure 4 illustrates the growth performance of this isolate in the modified liquid media containing either V^5+^ or Ni^2+^ or both at two different concentrations to highlight the difference of the toxic effect between the two metals. In general, the growth of the isolates decreased with the increases of the metal concentrations. The MWI-1 was able to significantly grow in the presence of V^5+^ at 100 mg/L [9 logCFU/mL] and 200 mg/L [8 logCFU/mL], while in the presence of Ni^2+^ MWI-1 could only grow at 100 mg/L. Concomitantly, Ni^2+^ toxicity was able to inhibit the growth in all the volume ratios. Statistical evidence revealed a significant difference (p<0.05) in terms of growth performance between the positive controls and those samples treated with Ni^2+^ while no significant difference (p>0.05) was indicated for MWI-1 treated with V^5+^. Another statistical analysis shows a significant difference for positive controls when compared with those samples treated with both metals concomitantly.

A general observation indicated that the MWI-1 isolate was more tolerant to V^5+^ than to Ni^2+^ in the modified liquid media (Table 4). The MWI-1 isolate could resist V^5+^ (MIC) up to 13.41 mM [approximately 700 mg/L] and only reached 5.39 mM [approximately 250 mg/L] in the presence of Ni^2+^ at pH 7. When exposed to test metals (V^5+^ and Ni^2+^) at acidic medium (pH 3), the results showed a decrease of tolerance ability of the isolate with 24 h-LC50 ranging from 5.89–6.87 mM for V^5+^ and 2.04–2.22 mM for Ni^2+^ and MIC ranging from 10.8–11.78 mM for V^5+^ and 2.56–2.73 mM for Ni^2+^. The tolerance or sensitivity of the MWI-1 isolate was also revealed by its ability to remove V^5+^ and Ni^2+^ at pH 7 (Table 4). In the presence of V^5+^ or Ni^2+^ separately (pH 7), the MWI-1 was able to remove up to 99.95% of 100 mg-V^5+^/L and 86.42% of 100 mg-Ni^2+^/L. When pH was adjusted at 3, the removal of both metals decreased to a range of 76.35–59.69 and 63.53–38.27 for V^5+^ and Ni^2+^, respectively. In consortium, Ni^2+^ toxicity appeared to disturb the removal of V^5+^, none of the metals (V^5+^/Ni^2+^) was removed at a percentage of over 30%, with the exception of V^5+^ (30.15%) that was removed at a ratio of 1∶1 [100 mg/L/100 mg/L]. The non parametric Kruskal Wallis test was performed to compare the rank sum of the tolerance rate of the bacteria between V^5+^ and Ni^2+^. The test was significant at a 0.05 significance level with a p-value of 0.0037. The rank sum of the tolerance level of the bacteria was higher in V^5+^ than Ni^2+^. Another statistical analysis revealed a significant difference (p<0.05) in terms of tolerance ability to Ni^2+^ between the two experiments (Experiment at pH 7 and pH 3), whereas no significant difference (p>0.05) in terms of V^5+^-tolerance was shown between the two experiments. This implies that at the pH (pH value 3) of the industrial wastewater (polluted environment), the isolate (MWI-1) ability to resist to Ni^2+^ decreases, while for V^5+^ there is no decrease in terms of tolerance ability when compared to the tolerance at pH 7.

**Table 4:** MWI-1 isolate tolerance limits (MIC and 24 h LC_50_) to V^5+^ and Ni^2+^, and removal ability in the modified liquid media (n = 5).

	Percentage removal (Single metal), pH 7		Percentage removal (Single metal), pH 3	
	100mg/L	200mg/L	100mg/L	200mg/L
V^5+^ [%]	99.23±0.57	94.6±2.19	76.32±4.29	59.69±2.15
Ni^2+^ [%]	82.42±3.43	43.67±3.76	63.59±6.71	38.27±1.65
	**Percentage removal (V5+/Ni2+, v/v) (mixed metals), pH 7**			
	**100/100 [mg/L]**	**200/100 [mg/L]**	**100/200 [mg/L] (v/v)**	
V^5+^ [%]	30.15±7.1	17.17±0.87	9.51±1.97	
Ni^2+^ [%]	23.96±2.6	11.48±1.48	6.04±2.82	
	**Tolerance limit at pH 7**		**Tolerance limit at pH 3**	
	**24h-LC50**	**MIC**	**24h-LC50**	**MIC**
V^5+^ [mM]	10.15±0.59	13.41±0.56	6.54±0.56	11.45±0.57
Ni^2+^ [mM]	3.68±0.49	5.39±0.5	2.11±0.16	2.67±0.1

During the course of the study, the ordinary linear regression and hierarchical linear model were used to compare the average percentage of metal removed by the bacteria between the three treatments, which are mixed, V^5+^ and Ni^2+^. Both adjusted and unadjusted models were fitted, and the treatments were found to be significantly different for both adjusted and unadjusted model. The treatments were found to be significantly different (p<0.05) (Table 5). In particular, V^5+^ and Ni^2+^ treatments were significantly different from mixed (p<0.05). Precisely on average V^5+^ used separately was removed 80.5%( 35.206 to 58.110) more than mixed, and nickel was removed 46.7% (35.206 to 58.110) more than mixed. Similar statistical observation was noted after adjusting for concentration and individual elements with on average V^5+^ removed 61.3%( 52.230 to 70.375) more than mixed, and Ni^2+^ removed 27.5.6% (18.405 to 36.550) more than mixed. This statistical evidence revealed that V^5+^ is less toxic then Ni^2+^, and there is interaction between the two metals which increase their toxicity and rending their mixture more toxic then individual elements.

**Table 5:** Unadjusted and adjusted hierarchical regression model-Percentage removal.

Covariates	A	B	C
V^5+^	80.48(69.54–91.42)	61.30(52.23–70.38)	69.61(65.03–74.19)
Ni^2+^	46.66(35.72–57.60)	27.48(18.40–36.55)	19.17(14.59–23.75)
200/100 mg/L		−12.73(−20.35–−5.109)	−12.73(−16.28–−9.185)
100/200 mg/L		−19.28(−26.90–−11.66)	−19.28(−22.83–−15.73)
100 mg/L		22.14(14.52–29.76)	38.75(33.73–43.76)
Metal Code		5.117*(−1.107–11.34)	5.117(2.221–8.013)
V*100 mg/L			−33.22(−40.31–−26.12)
Constant	16.39(10.92–21.85)	24.50(18.27–30.72)	24.50(21.60–27.39)

Confidence Interval in parentheses; Note: p value for all the parameters was less than 0.01.

* p<0.05, n = 30.

**A**: Main effect, **B**: Adjusting for concentration and individual elements, **C**: Adjusting for concentration, interaction between the treatment and the concentration, and individual elements.

Note: Each model includes the coefficient and their confidence interval in bracket.

#### Effect of pH on the tolerance limits of test organisms

To check the effect of pH on the tolerance limit of the isolate to V^5+^ and Ni^2+^, the MWI-1 inoculated in wastewater liquid media containing separately the test metal at a concentration able to kill the 50% of the test isolate after 24 h (LC_50_) and the experimental study was conducted at 30°C in a shaking incubator at a speed of 100 rpm. Table 6 shows an increase on percentage die-off rate over the decrease of pH throughout the study period. Furthermore, the percentage die-off rate of the isolate (MWI-1) also increased over time of exposure. When stressed with Ni^2+^, the percentage die-off rate of the isolate ranged from 39.25% (pH 8) to 85.15% (pH 1) after 24 h of exposure, whereas in the presence of V^5+^, the isolate die-off rate could not research 60% after 24 h of exposure.

**Table 6:** Percentage die-off rate of *Marinobacter* sp. MWI-1 stressed with V^5+^ and Ni^2+^ over various pH in wastewater liquid media (n = 3).

	Nickel				Vanadium			
pH	Day 1	Day 2	Day 3	Day 4	Day 1	Day 2	Day 3	Day 4
1	85.15	99.5	99.5	99.5	52.8	89.2	99.5	99.5
1.5	77.11	99.6	99.6	99.6	52.4	85.3	99.6	99.6
2	75.31	99.59	99.59	99.59	47.7	72.5	85	97.4
2.5	65.12	96.51	99.61	99.61	52.5	72.6	86.5	96.9
3	44.92	95.34	99.58	99.58	47.8	76.7	84.7	95.2
3.5	58.49	91.32	99.62	99.62	36.2	75.3	88.5	93.2
4	59.73	83.22	93.96	99.66	55.1	60.7	81.6	91.8
4.5	49.77	69.41	94.98	99.54	51.1	55.6	80.1	88.7
5	48.82	77.56	94.09	99.61	47.4	53	77.1	86.9
5.5	41.75	73.3	93.69	99.51	50.2	52.9	80	84.7
6	48.98	74.49	94.39	98.98	49.5	49.5	71.8	75.5
6.5	51.02	79.59	95.92	97.14	52.4	55.2	75	78.2
7	45.96	68.98	79.8	87.37	28.6	47.3	70.1	74.1
7.5	43.98	58.59	60.65	88.43	55.1	55.1	71.9	76
8	39.25	57.94	60.62	83.64	35.1	44.3	67.9	70.2

Subsequently, a statistical multivariate analysis was performed to check the difference between die-off rates at different pH-values when considering the type of test metals. Table 7 indicates that the effect of pH level on the die-off rate was significant at a 0.05 significance level. Specifically, an increase of 0.5 in the pH level decreases the die-off rate by 2.5% (−3.009 to −2.083). After adjusting for the type of metals, adjusting for the incubation period, adjusting for the type of metal and incubation period, significant difference (p<0.05) was also found between die off rates at different pH level. Similarly to the above, an increase of 0.5 in the pH level decreases the die-off rate by 2.5% (−3.009 to −2.083). However, when adjusting for the type of metal, incubation period and the interaction between the type of metal and the pH level was significant at a 0.05 significance level with an increase of 0.5 in the pH level decreases the die-off rate by 3% (−3.338 to −2.598). This implies that changes on pH level affect the growth of the isolate when exposed to V^5+^ or Ni^2+^ and this is also function on the incubation period. In addition, the statistical test also revealed that the optimal pH of the MWI-1 isolate in the modified wastewater liquid media ranges between 7 to 8 pH values.

**Table 7:** Unadjusted and adjusted hierarchical regression model-pH effect vs die-off rate, n = 30.

Covariates	pH_code	V	Day	Metal*pH level	Day*pH level	Constant
**A**	−2.546 (−3.009–−2.083)					81.64(69.28–94.00)
**B**	−2.546 (−3.009–−2.083)	−12.03(−16.03– −8.029)				87.66(82.99–92.32)
**C**	−2.546 (−2.814–−2.278)		22.18(19.86–24.49)			70.55(58.51–82.59)
**D**	−2.546(−2.814–−2.278)	−12.03(−14.35– −9.713)	22.18(19.86–24.49)			76.57(73.63–79.51)
**E**	−2.968(−3.338–−2.598)	−18.79(−23.54– −14.03)	22.18(19.92–24.44)	0.844(0.321–1.368)		79.95(76.40–83.49)
**F**	−1.769(−2.113–−1.425)	−12.03(−14.13– −9.930)	34.60(30.18–39.03)		−1.554(−2.040– −1.067)	70.35(67.06–73.65)
**G**	−2.191(−2.599–−1.783)	−18.79(−23.07– −14.50)	34.60(30.32–38.89)	0.844(0.373–1.316)	−1.554(−2.025– −1.082)	73.73(70.02–77.44)

Confidence Interval in parentheses; Note: p value for all the parameters was less than 0.01.

**A**: Model Main effect, **B**: Adjusting for metal, **C**: Adjusting for incubation period (Time), **D**: Adjusting for metal and time, **E**: Adjusting for metal, time and metal*pH interaction, **F**: Adjusting for metal, time and time* pH interaction, **G**: adjusting for metal, time, metal*pH interaction and and time* pH interaction.

Note: Each model includes the coefficient and their confidence interval in bracket.

#### SEM, FTIR and Molecular analyses

**Figure 5:**
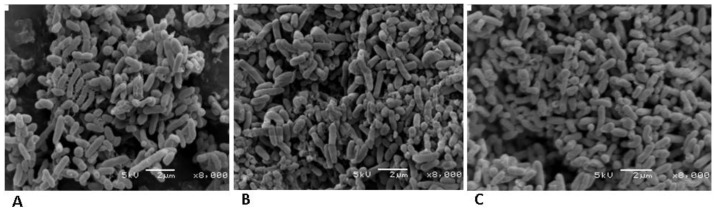
Scanning electron micrograph of strain MWI-1 grown on wastewater liquid media without test metal stressed (A), with vanadium stress (B) and with nickel stress (C).

To assess the effect of metal ions on cell morphology of MWI-1 in wastewater culture medium, a scanning electron microscopy (SEM) was used (Figure 5). The bacterial cells exposed to metal ions were significantly changed and showed major damage, characterized aglomeration and lyse of microbial cells when compared to isolate not exposed to metal ions. Microbial cells exposed to Ni^2+^ (Figure 5b) revealed more damage than those exposed with V^5+^ (Figure 5c).

**Figure 6:**
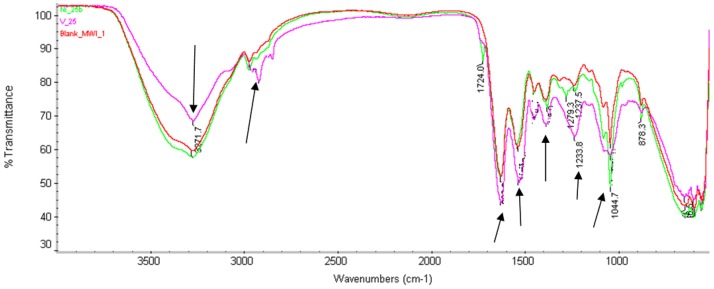
FTIR spectra of Marinobacter sp. MWI-1 before and after exposure to test metals.

The Fourier transform infrared spectroscopy (FTIR) analysis was perfomed to investigate the role of extracellular polymer in V^5+^ and Ni^2+^ bioadsorption. The FTIR spectroscopy of untreated and treated MWI-1 with V^5+^ and Ni^2+^ separately is shown in Figure 6. The result revealed the effects of metal ions (V^5+^ and Ni^2+^) on the functional group of membrane wall elucidating their biosorption onto the membrane. The bacterial biomass' spectrum (Blank) revealed different peaks representing amid group (C = 0, N-H, C-N), carboxylic group (C-0) and phosphate group (P = 0), reflecting the complex nature of the bacterial wall. A significant difference was found between untreated biomass and V^5+^-treated biomass, whereas the Ni^2+^-treated biomass did not show major difference when compared to the untreated biomass. The spectrum pattern of V^5+^-treated biomass showed changes of certain bands in the region of 870–1626.91 cm^−1^ and 2921.8–3271.7 cm^−1^ compared to untreated biomass. The spectrum pattern of Ni^2+^-treated biomass however showed changes in the region of 1044.7–1301.8 cm^−1^ compared to the untreated biomass' spectrum. This revealed that MWI-1 could adsorb more V^5+^ than Ni^2+^ in the liquid media.

**Figure 7:**
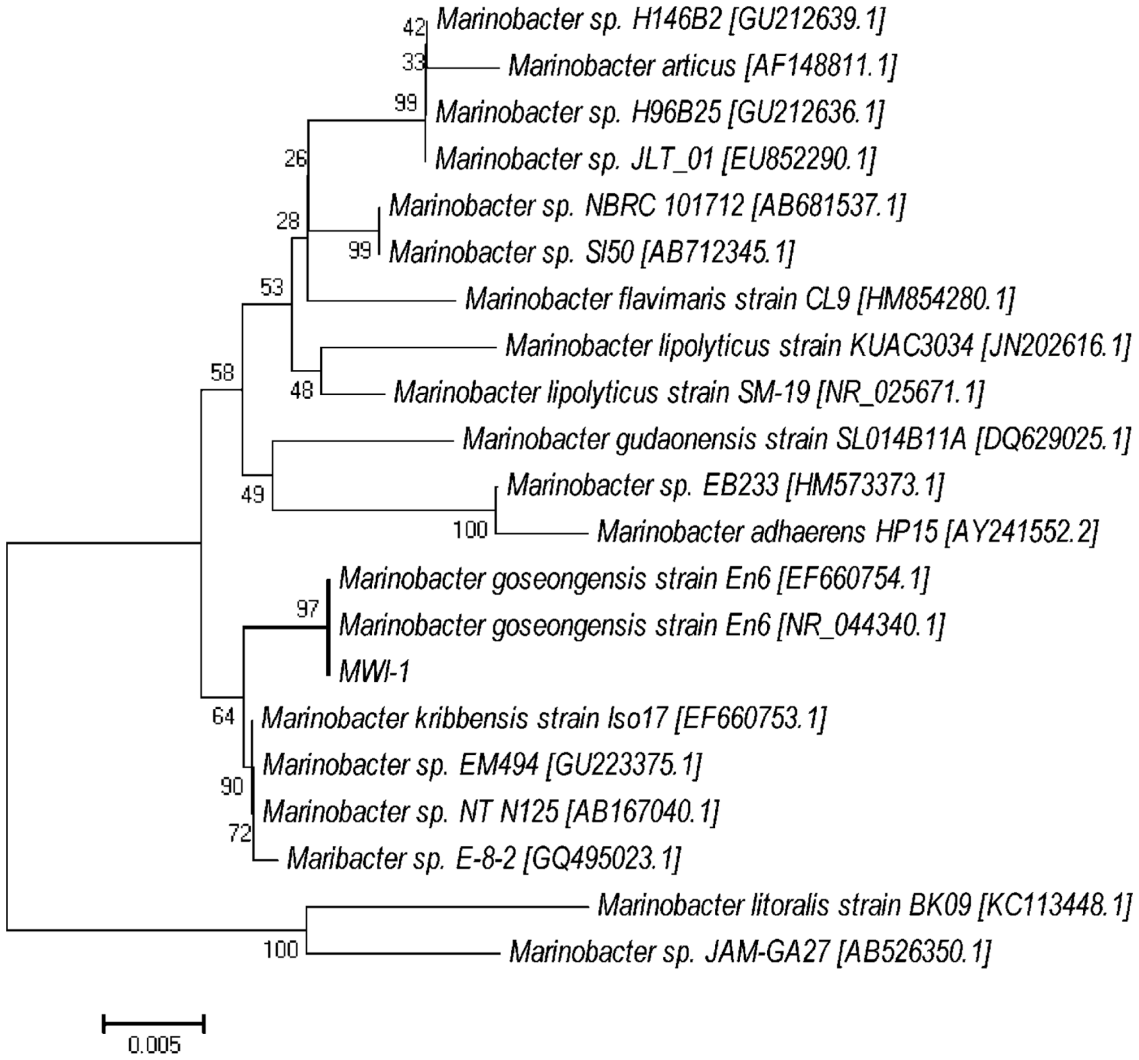
Phylogenetic tree using the neighbour-joining method, constructed and based on the bacterial 16S rRNA gene sequence detected in the present study along with similar sequences detected from the NCBI and RDP databases.

**Figure 8:**
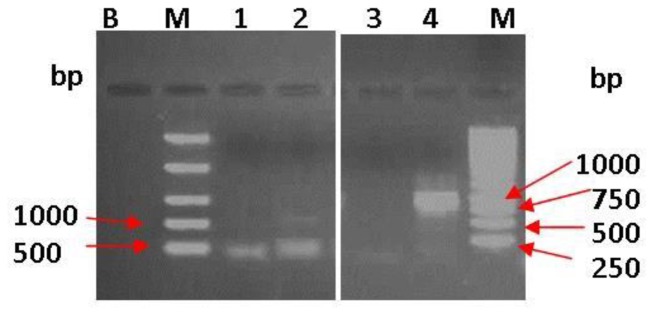
Agarose gel electrophoresis of PCR products of total genomic DNAs with primers targeting gene nccA (Lane: 4), van2 (lane: 3), smtAB (lane: 2) and cnrB2 (lane: 1). Lanes: M: DNA ladder (Marker) and B: Negative (No template DNA).

The phylogenetic analysis using the neighbour-joining method with a bootstrap value of 100 replicates indicated that an MWI-1 isolate belonged to the genus *Marinobacter* and was most closely related to *Marinobacter goseongensis* strain En6 [EF660754.1 and NR044340.1] at a similarity of approximately 97% (Figure 7). Molecular study investigated the resistance ability of the microbial isolates in a gene level, to check whether the heavy-metal removal ability of test isolate is linked to specific genes such as *nccA* (Ni, Co, Cd-resistance), *cnrB2* (Ni and Co-resistance), *van2* (V-resistance) and *smtAB* (gene encoding synechococcal metallothioneins) using the conventional PCR (Figure 8). The *smtAB* gene was targeted as it encodes the production of metallothionein involved in the resistance to several metal ions (such as Zn, Se, Cd, Hd, Ag, As, etc.) in several bacterial species. Of all the genes targeted in the gDNA of MWI-1 isolate, *nccA*, *cnrB2* and *smtAB* showed positive amplification. An amplified products of approximately 400 bp, 500 bp and 1141 bp revealing the presence of *cnrB2*, *smtAB* and *nccA* genes were reproductively detected, whereas, the vanadium-resistant gene *van2* was not found.

## Discussion

Mine water pollution negatively impacts the chemical and microbiological quality of both surface and groundwater [23]. Being a country with extensive industrialisation and major historical mine activities, water pollution by metal ions has emerged as one of the serious challenges currently faced by water service authorities in South Africa. As a result, the National Water Act of 1998 has been signed to make provision for the legal requirements, registration and authorisation to discharge wastewater into water sources [24]. Hence, this study firstly focused on the chemical characteristics of the vanadium mine located in Mpumalanga, South Africa. The results revealed that the mine water samples used in this study were highly acidic at a range below the permissible minimum limit of 5.5 pH unit set by the South African Water Act, No. 36 of 1998. If discharged into the receiving water bodies, this might be identified as a major factor in the speciation and diversity of microbial populations [25]. The electrical conductivities of the mine water varied significantly (p<0.05) and were found to be at a range above the maximum permissible limit of 250 µ/Scm for effluent discharged into the receiving water bodies [26]. Several other pollutants found in the mine water samples had concentrations which far exceeded the maximum recommended limits of 0.2 mg-Ni/L, 0.1 mg-Mn/L, 0.1 mg-V/L, 0.01 mg-Cu/L, 0.1 mg-Zn/L, 0.005 mg-Hg/L, 0.03 mg-U/L, 0.3 mg-Fe/L, 15 mg-NO_3_^−^/L, 10 mg- PO_4_^3−^/L and 75 mg-COD/L, prescribed by the UN-FAO, EPA and South Africa Water Act, No. 36 of 1998 [24], [27], [28], [29].

This study secondly focused on determining the bacterial diversity in the mine water samples and assessing their tolerance to high V^5+^ and Ni^2+^ concentrations in modified wastewater liquid media. With the use of a culture-independent method, a diverse bacterial population was found in the mine water samples as presented in Figure 1 and Table 3 with *Marinobacteria* (47.02%), indicating a predominance among other families. It is known that uncultured bacteria constitute the largest portion of the population in the environment [30]. In this study, uncultured bacteria were excluded and only approximately 15% of bacteria were unclassified. Although several studies have revealed evidence that free-living microorganisms exhibit non-random distribution patterns across diverse habitats at various spatial scales, the geochemical parameters also determine the type of microbial population and microbial diversity of a specific habitat [25], [31]. Therefore, it proved to be difficult to compare the present result on microbial diversity to those previously published on other habitats. Nevertheless, it has been reported that *Proteobacteria* are present in extreme environments [32]. A study by Raji et al. [33] revealed the predominance of *Proteobacteria* (53%) and other uncultured bacteria (41%) in deep mines in South Africa. Other studies on microbial diversity in hot water springs in South Africa, reported a predominance of *Proteobacteria*, *Bacteriodetes* and *Planctomycetes* at a percentage of 54.43%, 10.74% and 13.03%, respectively, with an abundance of *Xanthomonadales* belonging to the *Proteobacteria*. Another study conducted in China revealed that 60.62% of the microbial population in the mine water samples was affiliated to *Proteobacteria* phylum with a proportion of 3.10% being *Alphaproteobacteria*, 24.78% *Betaproteobacteria*, 31.41% *Gammaproteobacteria* and 1.33% *Deltaproteobacteria* [32]. Kuang et al. [25], when assessing the microbial diversity of acid mine drainage samples from southeast China, reported a predominance of *Proteobacteria*, with the *Betaproteobacteria*, which were linked to an acidic environment, at pH>2.4.

Due to their abundance, the *Marinobacter* genus of the *Proteobacteria* phylum was isolated in the present study and assessed for their tolerance to and removal of V^5+^ and Ni^2+^ in the modified liquid media. The isolate (*Marinobacter* sp. MWI-1) was firstly sequenced and found to be closely related to *Marinobacter goseongensis*. This *Marinobacter* sp. MWI-1 showed very good growth at 30°C in the HMC broth, while at 25°C and 35°C a prompt die-off was noted when inoculated in a metal-free media. Findings of this study corroborated to those of Roh et al. [17] who reported that the optimum temperature for *Marinobacter goseongensis* sp. nov. should be between 25°C and 30°C. When inoculated in modified wastewater liquid media, culture media containing V^5+^ and Ni^2+^, separately or combined and incubated at 30°C, reveals a significant growth (p<0.05) of the *Marinobacter* sp. MWI-1 in the media with V^5+^ when compared to the media with Ni^2+^. *Marinobacter* sp. MWI-1 was more tolerant towards V^5+^ than towards Ni^2+^ (Table 4). The toxicity of the test metals to *Marinobacter* sp. MWI-1 appeared to have a relatively negative effect on the metal-removal ability of the test isolate in the modified wastewater liquid media with V^5+^ indicating the highest level of removal regardless of the pH value of the culture media (Table 4). It has been reported that bacterial strains can be characterised as being tolerant towards metal such as Ni^2+^, if it is capable of expressing growth at concentrations higher than 100 mg/L of the metal [34]. This indicated that although the toxic effect of Ni^2+^ on the isolate when compared to the effect of V^5+^, the isolate was still tolerant towards the metal (Ni^2+^). However, the percentage removal decreased when both metals were concomitantly used revealing a synergistic action between the two metals. The tolerance ability of the test isolate towards Ni^2+^ was confirmed by a positive amplification of genes encoding the resistance of Ni^2+^, whereas the gene *van2* encoding vanadium resistance was not found. The present study could not provide sufficient evidence of *Marinobacter* sp. MWI-1 V^5+^ resistance ability. The tolerance to V^5+^ by the *Marinobacter* sp. MWI-1 could be explained by the presence of a gene (*smtAB*) encoding the production of mettalothionein which is a family of cysteine-rich proteins capable to bind metals and are suspected to be also involved in providing metal-resistant ability to bacteria [35].

The microbial species have been reported to be highly resistant to heavy metals and also having a very high ability to remove heavy metals. Zucconi et al. [36] stated that isolating microorganisms from extreme environments represent an appropriate practice to select metal-resistant strains that could be used for heavy metal removal and bioremediation purposes. Since most of the species of the genus *Marinobacter* have been reported to be halophilic, heterotrophic neutrophiles and living under extreme environmental conditions such as pH and high salinity, they have been isolated from several habitats such as seawater, petroleum refineries, oil-refineies, and so forth [37]. Due to their ability to grow under extreme habitats, strains found in *Marinobacter* could have the ability to remove metal in the environment. Researchers have reported that several strains of *Marinobacter* spp. such as *Marinobacter aquaeolei* have iron transport capabilities and are also capable of oxidising iron [38]. It has also been revealed that the abundance of genes involved in phosphonate metabolism in *Marinobacter* spp. could serve as binding agents for metals and furthermore be used as a heavy metal defence by these species [39]. According to Liao et al. [40], the *Marinobacter* species can be able to tolerate high concentrations of metal and metalloids. The authors revealed that *Marinobacter* sp. MnI7-9 isolated in a deep Indian sea could grow well at Mn concentration of 10 mM and also oxidize the metal at the same concentration.

Previous studies have well illustrated the importance of pH in microbial growth and activities which tend to be seen as one of the major limiting parameter for the performance of wastewater treatment systems [18]. In addition, acid-tolerant microorganisms are viewed as being beneficial for the treatment of highly polluted wastewater from the mines or industry [40]. However, by investigating the effect of pH variations on bacterial activity in the wastewater liquid media treated with V^5+^ or Ni^2+^ (at concentrations reaching their specific 24 h-LC_50_), the present study showed that the tolerance capability of *Marinobacter* sp. MWI-1 was significantly dependent on the pH and temperature of the wastewater liquid media (Table 4).

When assessing the biosorption of the test metal ions by biomass (Figure 7), it has been found that V^5+^ was more active on membrane wall implying high adsorption by *Marinobacter* sp. MWI-1 compared to Ni^2+^. Shifts in bands and peaks have also been reported by Lameiras et al. [41] and Tunali et al. [42] and these was reported to be indicative of bondage of functional groups (e.g. hydroxyl group and –NH stretching peak), indicative of Stretching of functional groups (C-N and aromatic-CH) and elucidating metal biosorption.

During the course of the study, a multivariate statistical analysis was performed supposing that the observations were dependent between each other. For each parameter, data analysis was repeated assuming the observations are independent and the results were the same as for the dependent case, with differences only in the width of the confidence interval.

In conclusion, the MWI-1 isolate (*Marinobacter* sp. MWI-1 [AB793286]), closely related to *Marinobacter goseongensis*, demonstrates high tolerance to both V^5+^ and Ni^2+^. This indicates that mine water is a reservoir of novel microbial species which can sufficiently be used for the removal of heavy metals in highly polluted effluents. The tolerance and removal ability of the isolate (*Marinobacter* sp. MWI-1) was found to be pH and temperature dependent with 7-8 pH values and 30°C as an optimum, respectively. Further studies on geochemistry and microbial diversity need to be conducted to unveil how the chemistry of the effluent from the vanadium mine in South Africa can affect the microbial diversity of the environment. Furthermore, studies carried out on microbial diversity from extreme environments such as mine water are needed in order to isolate novel hyper-tolerant microbial species for heavy metal removal. The results in the present study constitute one of the first regarding the bacterial diversity of mine water samples from vanadium mine in South Africa.
